# Occupational health safety in aquaculture: A case study on semi-intensive shrimp farmers of Bangladesh

**DOI:** 10.1371/journal.pone.0315075

**Published:** 2025-02-13

**Authors:** Md Mostafizur Rahman, Md. Khaladur Rahman Shohag, Md. Ridwanul Islam, Md Shamim Hasan, Jinat Ara Nasrin, Mst. Muslima Khatun, Sudip Debnath, Md. Moshiur Rahman, Edris Alam, Khawla Saeed Al Hattawi, Md Kamrul Islam, Abu Reza Md. Towfiqul Islam

**Affiliations:** 1 Faculty of Arts and Social Sciences, Department of Disaster Management & Resilience, Bangladesh University of Professionals, Dhaka, Bangladesh; 2 Fisheries and Marine Resource Technology Discipline, Khulna University, Khulna, Bangladesh; 3 Department of Geography and Environmental Studies, University of Chittagong, Chittagong, Bangladesh; 4 Faculty of Resilience, Rabdan Academy, Abu Dhabi, UAE; 5 Department of Civil and Environmental Engineering College of Engineering, King Faisal University, AlAhsa, Saudi Arabia; 6 Department of Disaster Management, Begum Rokeya University, Rangpur, Bangladesh; CIFRI: Central Inland Fisheries Research Institute, INDIA

## Abstract

The study examined health safety issues among semi-intensive shrimp farmers in southwestern Bangladesh. The study assessed semi-intensive shrimp farmers’ knowledge, attitudes, and practices (KAP) on health and safety during their farm activities. The KAP model examined working circumstances, exposures, health complaints, diseases, current health status, and job satisfaction. Face-to-face interviews were conducted with 158 semi-intensive shrimp farmers from Bangladesh’s Khulna, Satkhira, and Bagerhat districts. Type-based data analysis was done. We used Spearman’s rank correlation and multiple linear regression. Only 35% of respondents said that they knew the health safety of chemicals and pesticides used in semi-intensive shrimp farms and that safety training is needed, and all farmers wanted to attend when available. At least 74% of farmers used masks when handling pesticides, fertilizers, and chemicals. 40.50% of farmers used protective clothing, 91% cleansed their hands, legs, and face after each day’s work, and 94% felt that sanitization reduces pesticide, fertilizer, and chemical pollution. A significant positive correlation coefficient was established in KAP. Education significantly increased knowledge. Practices varied with health and safety training. Results suggest that attitudes determine practices, where knowledge is significantly associated with attitudes. A comprehensive health safety and hygiene training program for all shrimp farmers should be developed to raise awareness, reduce illness outbreaks, and preserve healthy living.

## Introduction

Bangladesh has a substantial agricultural boom in response to the increasing need for food, given its status as one of the most densely populated countries. Its robust fisheries sector significantly contributes to Bangladesh’s economy and food security. Aquaculture prowess is exemplified by Bangladesh’s third-place global ranking in fish production [1,2]. Bangladesh’s shrimp production and exports rank eighth and seventh globally, making substantial contributions to the country’s employment, socioeconomic development, and economy [1,3–5]. Southwestern coastal Bangladesh is well-suited for shrimp aquaculture because of its advantageous biophysical conditions, culture conditions, and capacity [6,7]. This coastal area of Bangladesh has historically served as the primary center for shrimp and prawn production [8].

Shrimp farming in Bangladesh can be classified broadly into three distinct types based on the inputs utilized: (1) Extensive culture, wherein the growth of the shrimp is entirely reliant on naturally occurring organisms present in the ponds; (2) Semi-intensive culture, wherein growth is enhanced through the application of fertilizer, natural productivity, and a small amount of artificial diet. Additionally, semi-intensive systems often utilize aeration equipment to maintain adequate oxygen levels and optimize productivity; and (3) Intensive culture, wherein the shrimp predominantly obtain nutrients from artificial feeds [9,10]. Therefore, by approaching shrimp farming in a balanced manner, semi-intensive farming balances intense and extended techniques. In shrimp farming production, both semi-intensive and intensive models are widely used. These systems require juvenile shrimp, regular feeding, and artificial management, including applying chemicals or fertilizers. However, the semi-intensive model balances natural productivity and human intervention, using lower inputs than intensive farming while incorporating artificial feeding and aeration techniques [11]. Studying the semi-intensive model is crucial as it allows for optimizing resource use and sustainability, which is particularly important for regions with limited inputs and needing cost-effective production methods. In semi-intensive shrimp farming systems, the occurrence of diseases is generally lower compared to intensive systems. It is largely because such systems focus on maintaining better water quality and using fewer chemicals, except during key stages like initial or final pond disinfection. Probiotics are often employed during farming to enhance water quality, promote healthier environments, and prevent disease outbreaks. Probiotics play a critical role by improving the shrimp’s gut health and non-specific immune responses, reducing the reliance on antibiotics or harsh chemicals for disease management. These biological agents also help regulate the microbial ecosystem in shrimp ponds, contributing to better water quality and limiting the proliferation of harmful pathogens [12,13]. Additionally, semi-intensive systems offer a sustainable alternative by utilizing natural resources more efficiently and minimizing environmental degradation caused by the overuse of chemicals in more intensive shrimp farming models [14]. Thus, semi-intensive shrimp farming methods have gained popularity in the southwestern region of Bangladesh [15]. This farming practice has significantly increased shrimp yields and profitability and has a significant economic impact in Bangladesh [16,17]. However, the study underscores the ecological ramifications, emphasizing the imperative for sustainable methodologies [18]. It is essential to conduct research related to occupational health and safety in shrimp farming. It includes exploring common hazards shrimp farmers face, such as chemical exposure, ergonomic risks, and injuries from handling equipment and shrimp. Research shows that personal protective equipment like gloves and boots can significantly reduce injury risks for shrimp farm workers, but awareness and accessibility of these tools remain inconsistent [19]. Further investigation into the global state of aquaculture workers’ health shows that many shrimp farmers are exposed to various health risks, including respiratory issues from chemicals and musculoskeletal injuries from repetitive tasks [20]. Improved occupational health and safety measures and increased health and safety training can help mitigate these issues, enhancing farmer safety and overall farming productivity.

Bangladesh’s shrimp industry is a significant economic sector characterized by expanding job possibilities, export potential, and demand. However, domestic and international production and market uncertainties challenge this sector [8]. As a result of the unplanned spread of shrimp and prawn farming systems caused by frequent modifications to farming techniques, social and ecological problems have arisen in the region [7,21]. In contrast to its substantial fish output, Bangladesh has been grappling with a substantial decrease in shrimp production, which has had a detrimental effect on its shrimp industry. For instance, a considerable decrease in productivity throughout the region, attributable to various variables, causes an annual reduction in shrimp exports from Khulna (one of the primary shrimp production districts in the southwestern part of Bangladesh) [22]. A decline in the supply of disease-free fry, a reduction in the source of brackish water, viral outbreaks, and farmers’ reluctance to embrace new techniques have all contributed to a decline in shrimp output in Khulna [22–24]. The lack of modern, efficient policies tailored to the needs of the shrimp industry has hindered its competitiveness and development [25]. Global markets increasingly require detailed traceability for food products. Bangladesh’s shrimp industry lacks robust digital traceability systems, making it difficult to meet international standards and affecting export opportunities [26]. The presence of harmful chemical residues in shrimp muscle due to improper farming practices and lack of regulations further hampers export potential [27]. Countries like India and Vietnam have outperformed Bangladesh in shrimp exports due to better marketing, technology, and branding strategies [28]. Nevertheless, prior research has demonstrated substantial prospects for enhancing shrimp production in Bangladesh through intensification and more effective management strategies [9,29].

Substandard planning and management, along with a lack of enforcement of existing regulations, have contributed to the adverse effects of shrimp farming [18]. Unregulated and inadequately coordinated has been the increase of farmland [30]. The shrimp industry is constantly grappling with challenges primarily attributable to the escalating prevalence of diseases and the absence of governmental support: improved management practices, accessible seed, high-quality water and soil, and high-quality feed are imperative for the establishment of sustainable farms that are effectively managed [18]. As a result of increased stocking densities and feeding rates, the intensification of production systems has raised the danger of illnesses and caused detrimental changes in water quality. A variety of illnesses induced by viral and bacterial infections in shrimp farms in Bangladesh have been documented in many studies [31,32]. It is widely known that biosecurity and health management strategies at the farm level are crucial for mitigating the effects of diseases [18,33–35]. A diverse array of chemical and antibacterial compounds are commercially accessible to aid in preventing and treating diseases affecting several fish species, including shrimp, prawns, crabs, and others [32]. One significant limitation is that treatments are occasionally carried out without regard for the well-being of the shrimp farmers or environmental and food safety concerns [33]. Therefore, the overall lack of awareness among Bangladeshi farmers regarding occupational health consequences has become a significant worry regarding their health and safety. It may harm the health of semi-intensive shrimp farmers, who often fail to practice safety precautions while at work. Furthermore, inadequate management can lead to the accumulation of residues of potentially hazardous substances, such as antimicrobials or pesticides, in organisms that have been treated. This accumulation risks consumers and can cause complications in the marketing and export of aquaculture products [36].

Aquaculture has a multitude of diverse work risks [19,37–39]. The death and injury rates are significantly higher than in other sectors. Aquaculture workers around the globe have heightened vulnerability to occupational illnesses and injuries, with the associated hazards frequently being unreported [37]. The diversity of hazards, injuries, illnesses, and worker protection rules indicates the variations in production sizes, species cultivated, and operational activities throughout different locations. Studies have urged a global commitment to workplace safety and health concerns in aquaculture [19,33,37,40]. In addition, the International Labor Organization is formulating a code of conduct on "employment in the fishing industry" [41]. Bangladesh exports fish and fishery products (including shrimp) to several Asian, European, and North American countries. The breach of the Code of Conduct might have significant repercussions for the country’s export chain.

Poor working conditions can reduce the quality of processed shrimp, ultimately affecting export potential [42]. Ensuring the health of shrimp farmers is equally as important as ensuring high-quality shrimp output. Bangladesh’s shrimp output may increase if the health and safety of shrimp farmers are preserved by responsible farm management. Furthermore, this will bolster the global reputation of Bangladeshi shrimp and allow it to continue its position as an industry leader. Thus, this study can help improve the shrimp export industry by improving occupational health and safety measures and boosting productivity. By ensuring better health and safety standards, workers can operate more efficiently, leading to higher-quality products, fewer losses due to illness or injury, and improved compliance with international export standards. Enhanced worker well-being will also likely translate into higher production outputs from processing plants, thus increasing exports [42]. While no specific case studies have been provided, international standards and general findings indicate that a healthier workforce yields better production quality. Thus, improving health safety measures will likely have a direct, positive impact on the shrimp industry in Bangladesh [27].

Utilizing a knowledge, attitude, and practice (KAP) survey model, this study aims to ascertain the health and safety conditions of semi-intensive shrimp farmers in the southwestern area of Bangladesh (districts of Khulna, Satkhira, and Bagerhat). This study further analyzed the relation between sociodemographic information and KAP status to ascertain the degree to which farmers are focused on health and safety. The KAP model originated in the 1950s and has been widely applied to assess the degree of knowledge exhibited by survey participants for a given subject [43–46]. The convenience of this survey approach stems from its straightforward structure, accurate outcomes, and concise report [47]. It is also hypothesized that it illustrates the interaction between the KAP domains of respondents [48]. Communities could better prepare for and respond to public health emergencies if the KAP level were to be determined. Such research may contribute to developing interventions to promote desired behavioral changes [49]. Consequently, this research may contribute to developing policies and initiatives to mitigate health hazards among shrimp farmers in Bangladesh. Furthermore, the findings of this research may distribute vital information to governmental and non-governmental groups throughout the globe so that they may formulate initiatives to mitigate health risks among farmers, with a specific focus on those employed in semi-intensive shrimp farms.

## Methods

### Study area

**Fig 1:**
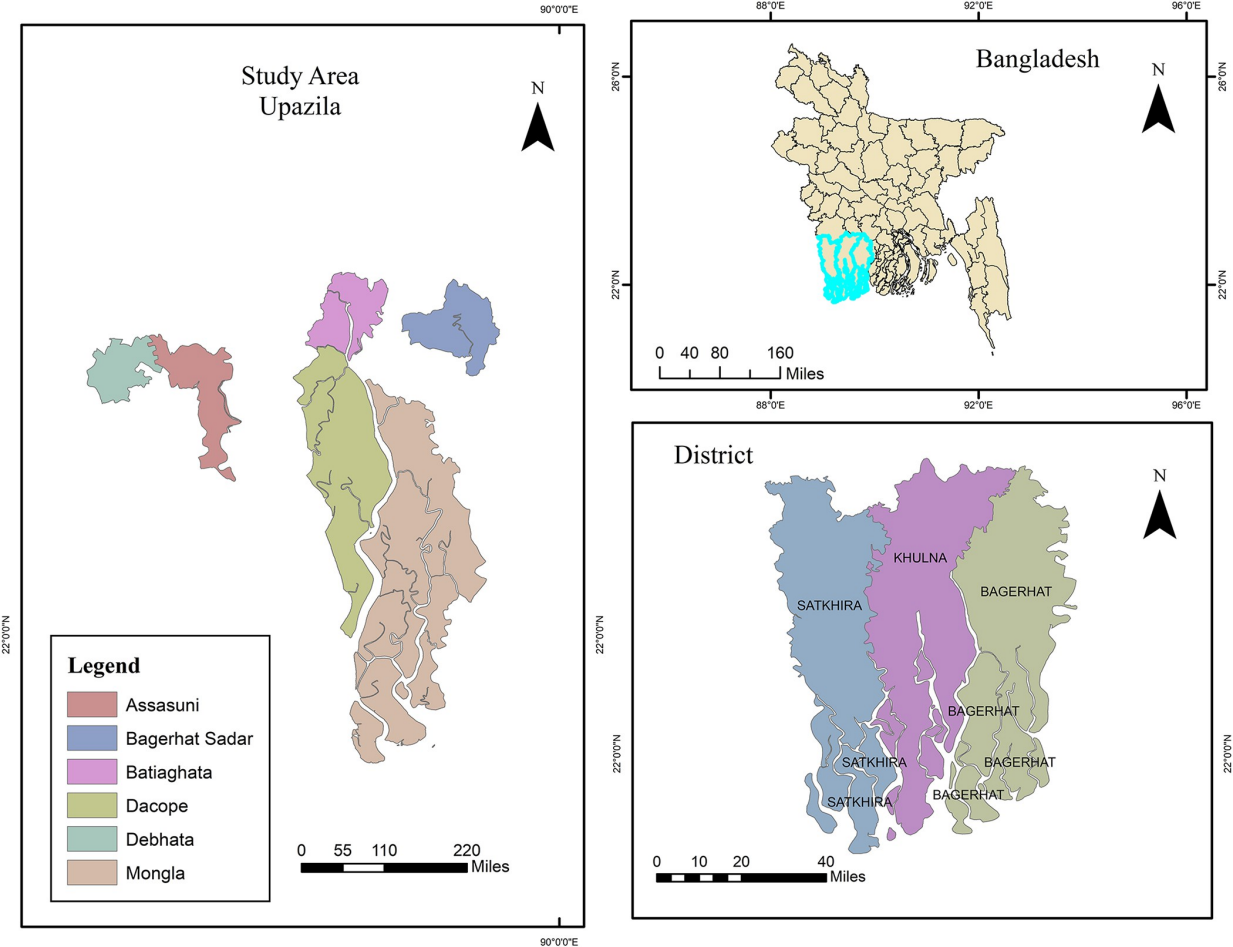
Study area (Base map and data from OpenStreetMap and OpenStreetMap Foundation [51]).

The southwest region of Bangladesh was chosen for this cross-sectional study depending on the existence of semi-intensive shrimp farms in the area. Under the three separate districts (Satkhira, Khulna, and Bagerhat), we selected six upazilas (Batiaghata, Dacope, Bagerhat Sadar, Mongla, Assasuni, and Debhata) (Fig 1). Semi-intensive shrimp farming is common in southwestern Bangladesh, especially along the coast. According to earlier research, shrimp farmers in this area often use semi-intensive monoculture techniques [15,50]. We aimed to examine the health issues posed by semi-intensive shrimp farmers using KAP. We hypothesized that there could be unmet demands in the health safety-related responses utilizing KAP’s three sections. Our secondary goal was to examine the relationship between several independent variables and health safety responses. To do this, we performed descriptive and inferential statistical analysis on the state of KAP and the factors that affect these farmers’ health and safety. We only looked at adult farmers (18 years and above). Data from the sample was collected through a face-to-face survey.

### Survey tools

The local language, Bengali, was used to conduct the survey. The KAP on health and safety in shrimp farms served as the basis for the survey’s content development. Our draft questionnaire was developed using literature reviews [52–55] and expert opinions. The final questionnaire was developed after completing a pilot survey and assessments by subject-matter experts. The results from the pilot survey were explicitly excluded from the final analysis to ensure the transparency of the research methodology. More than 0.70 was determined for Cronbach’s alpha. A questionnaire’s internal consistency is deemed reliable if its Cronbach’s alpha is greater than 0.60 [56,57]. The questionnaire was divided into three sections. In the first section, we addressed a few basic sociodemographic queries (age group, gender, marital status, education, occupation, monthly income, location, etc. of the respondents). We then included important health-related statements in the second section. The participant data from these two sections served as an independent variable in this study. We speculated that these factors impact each of the three KAP aspects. Finally, KAP-based responses about health and safety were provided. There were 32 items in the KAP section (08 items for knowledge, 13 for attitude, and 11 for practices). The study assigned a value of 1 to the correct answer, 0.50 to the neutral response, and 0 to the incorrect answer. If the response was valid, it was regarded as accurate (i.e., supported by current literature and expert reviews). For instance, in the case of knowledge, there was an item, “I know that I should wear a face mask when using chemicals, fertilizers, and pesticides”. If they agree, we could consider it a correct response supported by a previous study [58].

### Data management

The KAP model survey was conducted from 8^th^ August to 17^th^ October of 2022. Initially, we obtained an available list of semi-intensive shrimp farms in the designated regions from district-level fisheries authorities. The shrimp farmers from the farms were then chosen based on convenience (considering availability to reach). Thus, we followed a nonrandom sampling technique. A total of 158 semi-intensive shrimp farmers agreed to participate in our survey. The final analysis therefore comprised 158 respondents (Bagerhat Sadar = 18 and Mongla = 30 under Bagerhat district, Dacope = 30 and Batiaghata = 30 under Khulna district, Assasuni = 24 and Debhata = 26 under Satkhira district).

We employed the ’R’ program, version 4.2.2 [59], for statistical analysis. Descriptive statistics were calculated where appropriate. The average score was established to keep the analysis on the same scale. To estimate the section’s overall score, the scores for each item in the section were added together. Next, the result was divided by the item number in the section to get the average score (Eq 1). For instance, in the case of the knowledge section, total score obtained from 8 items were divided by the total number of items (08). Finally, Eq 2 was used to get the total KAP score. As a result, the KAP score was calculated using the same scale for all three portions (0–1 score range).

$$average\;score\;of\;section=\frac{\sum score\;in\;items}{\sum item\;number}$$ 1

$$KAP\;score=\frac{\sum average\;score\;of\;sections}{\sum section\;number}$$ 2

The correlation in the KAP domain was obtained by calculating Spearman’s rank correlation. In addition, we have employed the Wilcoxon signed-rank test to estimate significant differences in KAP domain. Finally, we utilized the multiple linear regression model to evaluate the relationships between the independent variables (health safety-related information and sociodemographic data such as gender, age group, education level, training in health safety, health safety rating in current occupation, and toilet facilities) and dependent variables (KAP sections regarding occupational health and safety for shrimp farmers). Multiple linear regression analyses only included significant variables from univariate linear regression. For instance, gender was found to be significantly associated with knowledge. Thus, we have added gender as one of the independent variables in the multiple regression analysis model, where knowledge was the dependent variable. We have checked the linearity to meet the assumptions of multiple linear regression analyses. In addition, we used Q-Q plots to confirm the normality of residuals.

### Ethical issues

This study has been approved by the ethical clearance committee of Khulna University, Bangladesh (Reference number: KUECC-2021/01/03). We took verbal consent before each questionnaire survey. However, consent was documented before each interview with the participant’s witnesses. There was no incentive to participate in the survey.

## Results and discussion

### Sample profile

Males (98%) and young adults (26–35 years old) make up the majority of the sample (Table 1). Almost everyone who responded was married (82%). Some of them have completed primary school or higher (90%). Around 56% made over 10,000 Bangladeshi takas (BDT) (100 BDT approximately equals $1) monthly. Of them, about 43% had 6–10 years of job experience.

**Table 1:** Sociodemographic characteristics of shrimp farmers (*n* = 158).

Category	Subcategory	*n* (%)
Gender	Male	155 (98.10)
	Female	3 (1.90)
Age Group (years)	18–25	47 (29.70)
	26–35	77 (48.70)
	36–45	30 (19.0)
	>45	4 (2.50)
Marital Status	Married	130 (82.30)
	Unmarried	28 (17.70)
Education level	>Primary	115 (72.80)
	Primary	29 (18.40)
	None	14 (8.90)
Monthly Income	5000–10000 BDT	70 (44.30)
	>10000 BDT	88 (55.70)
Working Experience?	<5 years	30 (19)
	6–10 years	69 (43.30)
	>10 years	59 (37.40)
Area	Bagerhat	48 (30.37)
	Khulna	60 (37.97)
	Satkhira	50 (31.65)

### Health safety-related status

Table 2 displays the aspects relating to safe health. Of the shrimp farmers, 83% had received training in health and safety. They mostly relied on friends and relatives as sources of knowledge about precautions. 92% of respondents said they were in good health during the study. Only 8% of them said they had a medical condition. 96% of them rated safe regarding health safety in their current occupation. Over 85% of respondents said their workplace had pucca (cement and brick) sanitary facilities.

**Table 2:** Health safety-related features of shrimp farmers (*n* = 158).

Category	Subcategory	*n* (%)
Are you trained in health safety?	Yes	131 (82.90)
	No	27 (17.10)
Which media do you use most for information related to precautionary measures?	University	2 (1.30)
	Internet	13 (8.30)
	National and Local Authority	10 (6.30)
	Print Media	0 (0.00)
	Electronic media (TV, Radio)	10 (6.30)
	Social Media	4 (2.50)
	People (Community, Family Members)	119 (75.30)
	
How can you rate your current health status?	Good	145 (91.80)
	Moderately Good	12 (7.60)
	Poor	1 (0.60)
Do you have any diseases of the following (diabetics, heart disease, back pain, skin disease, pneumonia, kidney problems, etc.)?	Yes	13 (8.20)
	No	145 (91.80)
How can you rate your health safety in your current occupation?	Safe	152 (96.20)
	Moderately Safe	5 (3.20)
	Unsafe	1 (0.60%)
Toilet facilities	Open Space	6 (3.80)
	Semi-pucca	16 (10.10)
	Sanitary/pucca	136 (86.10%)

### Health safety-related knowledge

Table 3 displays the shrimp farmers’ knowledge of safe health. Most respondents, 70%, said they were aware of safe health. Workers in shrimp farms are susceptible to various occupational injuries, highlighting the need for enhanced occupational health protocols in the industry [15,37]. It is suggested that the government and farm owners prioritize the occupational health and safety of workers in shrimp farms. Identification of hazards, instruction, and training are crucial for the health and safety of those who work in shrimp farms [19]. When utilizing chemicals, fertilizers, and pesticides on farms, a large percentage of them (75%) said they were aware that they should use face masks. Almost 92% knew they should exercise caution when using pesticides and fertilizers. Furthermore, sanitization before and after employing chemicals was known to more than 80% of them. Sanitization plays a vital role in safeguarding shrimp farmers from the health hazards associated with chemical use in aquaculture. Before using chemicals, proper sanitization of equipment and farm areas ensures no harmful residues or pathogens are present, reducing the risk of accidental chemical exposure due to reactions with contaminants [60]. This step is essential for minimizing the chances of inhalation, skin contact, or ingesting harmful substances, which could lead to acute or chronic health issues among farmers. After chemical application, thorough cleaning prevents the buildup of toxic residues that could pose long-term health risks to farmers through repeated exposure. Persistent chemical residues on equipment, ponds, or farm structures may lead to skin irritation, respiratory problems, or other health complications [61]. Post-chemical sanitization ensures the work environment remains safe, reducing occupational hazards and supporting farmers’ well-being. Therefore, implementing effective sanitization practices before and after chemical use is crucial for environmental and shrimp health and protecting shrimp farmers from potential occupational health risks. Nevertheless, only 35% of respondents knew enough about healthy living. This finding aligns with a 2016 research on Bangladeshi shrimp farms [33,62]. They found out that most farmers are uninformed and unconcerned regarding the potential long-term health repercussions of the pesticides employed on their farms. Furthermore, a significant proportion of the disinfectant packaging lacked sufficient textual health warnings on the product labels. Additionally, this research revealed that around 86% of respondents came into direct skin contact with chemical-containing substances and water through hand-handling [33,62]. Notably, this percentage was greater among small and large farm farmers [33,62]. Additionally, it was shown in previous studies that 58% of the surveyed farmers utilized protective equipment, namely masks, and polythene, when handling chemicals [33,62]. When mixing antimicrobials into feed with their bare hands, the majority of small-scale and nursery farmers had direct skin contact with chemicals, according to another study on occupational health risks associated with Pangasius catfish (*Pangasianodon hypophthalmus*) aquaculture in the Mekong Delta, Vietnam [63]. A study found a significant discrepancy in the use of protective equipment, particularly masks and gloves, between large-scale and small-scale shrimp farmers [63]. While all large-scale farmers reported consistently using protective gear when handling chemicals, only 40–50% of small-scale farmers did so. This difference may stem from several factors. Economic constraints could limit small-scale farmers’ ability to afford protective equipment. Additionally, limited access to safety education or training might result in a lack of awareness regarding the importance of personal protective equipment (PPE). Furthermore, accessibility issues, such as the availability of PPE in rural areas, may also play a role in this disparity. Understanding these barriers is crucial for designing targeted interventions to improve safety practices among small-scale farmers. Face masks and other personal protection equipment should take precedence when dealing with chemicals, fertilizers, and insecticides. Persistent pesticide exposure without appropriate protective gear may result in adverse health effects for farm laborers [64]. Furthermore, task-specific recommendations for masks are needed, given that pesticide labels frequently delineate the requisite level of protection [65]. About 80% of the farmers were informed that contaminated shrimp should be avoided. In shrimp farms in Bangladesh, viral and bacterial infections have been implicated in a variety of illnesses, according to several studies [32,34].

**Table 3:** Knowledge, attitude, and practices (%) regarding health safety (*n* = 158).

Section	Items	Agree	Disagree	Neutral
**Knowledge**	I am aware of health safety	69.60%	3.20%	27.20%
	I know that I should wear a face mask when using chemicals, fertilizers, and pesticides	74.70%	5.70%	19.60%
	I know that I should be away from the farm if I have any diseases (diarrhea, dysentery, cholera, flu, etc.)	95.60%	0.60%	3.80%
	There are some side effects of using fertilizers/chemicals	94.90%	0%	5.10%
	If I don’t follow precautionary measures, chemicals and fertilizers may be detrimental/harmful to my health	92.40%	0%	7.60%
	Sanitization is important before and after using chemicals	84.20%	3.20%	12.70%
	I have proper knowledge about health safety	34.80%	41.80%	23.40%
	Diseased shrimp must be avoided	79.70%	5.10%	15.20%
**Attitude**	I am aware of health safety	94.30%	0%	5.70%
	I should take special/strict safety while using raw foods, chemicals, and pesticides	91.80%	0.60%	7.60%
	Shrimp feed must be kept in a dry place	96.80%	0%	3.20%
	Training in hygiene practice is necessary	90.50%	0.60%	8.90%
	Training in sanitation practice is necessary	85.60%	3.20%	11.40%
	I will participate in these kinds of training	84.20%	10.10%	5.70%
	People who become sick should not be involved in working till they become well enough to work	96.80%	0.60%	2.50%
	Shrimp kept at room temperature can be contaminated	96.20%	0.60%	3.20%
	I should have proper safety while preparing food	85.40%	4.40%	10.10%
	Washing hands after using chemicals is necessary	99.40%	0%	0.60%
	The inhaling of smoking particles of chemicals and fertilizers is injurious/detrimental to health	93%	1.30%	5.70%
	Consuming infected shrimp is dangerous	87.30%	4.40%	8.20%
	Continue my profession without safety is unhealthy	59.50%	24.10%	16.50%
**Practice**	I am aware of health safety	40.50%	34.80%	24.70%
	I clean my hands, legs, and face after finishing each day’s job	91.10%	0.60%	8.20%
	I consume shrimp from our farm	90.50%	5.10%	4.40%
	I keep shrimp feed in a dry place	98.10%	0%	1.90%
	I take any proper safety while in contact with raw feeds	87.30%	5.10%	7.60%
	I wash my hands before eating foods after using chemicals/fertilizers/feeds	96.80%	0.60%	2.50%
	I wash my hands before and after using chemicals/fertilizers/feeds	85.40%	7.60%	7%
	I clean transportation materials and vehicles with clean water after using chemicals, fertilizers, and pesticides	96.20%	0%	3.80%
	I follow trusted information to protect my health from any hazards	97.50%	0.60%	1.90%
	I inform others when I become infected and request a break to prevent transmission of the disease	97.50%	0%	2.50%
	I don’t consume infected shrimp	86.10%	2.50%	11.40%

### Health safety-related attitude

In addition to knowledge, attitude is a crucial determinant that can impact health safety behavior and practice, reducing the incidence of illnesses associated with chemicals and pesticides. Sanitization might lower the danger of contamination from pesticides, fertilizers, and chemicals, according to 94% of farmers (Table 3). Additionally, they support implementing stringent safety measures while handling raw foods, chemicals, and pesticides. These results reflect their degree of awareness, with the majority being aware of the adverse impacts associated with the use of fertilizers and pesticides. Furthermore, it underscores the significance of adhering to rigorous precautionary protocols while utilizing chemicals and fertilizers in farming [19,32–34,55,62]. Their high degree of knowledge may have prompted these favorable attitudes. According to research, knowledge is critical in influencing positive attitudes [66]. In several fields, including health, it has been found that information influences attitudes and subsequent conduct [67,68]. Information’s extent, significance, and intricacy impact the congruence between attitudes and actions. Conversely, one study posits that despite farmers seemingly possessing a comprehensive understanding of health and safety, favorable attitudes are crucial for effectively implementing these practices on the farm level to prevent illness transmission [69]. Consequently, similar to their degree of knowledge, individuals hold favorable attitudes about avoiding the consumption of contaminated shrimp. Training programs play a critical role in bolstering the expertise and competencies of shrimp farmers, making a substantial contribution to the sustainability and well-being of shrimp farming methodologies. A significant proportion of the participants in our research concurred that training is necessary to enhance their understanding of shrimp farming and ensure each individual’s health and safety. Furthermore, they have a favorable disposition towards such training programs. In addition, the study reveals that news media, nearby farms, and aquaculture extension training served as information sources on protective measures [33]. A prior research revealed that none of the farms situated in the southwestern part of Bangladesh has either a health management plan or illness diagnosis facilities [33]. Additionally, it was shown in that study over 50% of the farmers surveyed had participated in one or more short-term aquaculture training courses. However, illness diagnoses were not included in the course. As a whole, 88% of farmers surveyed reported being impacted by diseases that originated in cultivated shrimp or prawn [33]. Furthermore, it was discovered that farmers acknowledged receiving instructions on properly handling chemicals and being informed of the health risks associated with such practices. Nevertheless, they could not articulate suitable protocols for chemical safety and the risks involved, indicating a potential requirement for enhanced educational initiatives and training programs [33]. Regarding hand hygiene, most shrimp farmers agreed that cleaning their hands after handling chemicals and pesticides was important. The present research result is consistent with the findings made in a previous study, which found that almost 97% of farm workers felt handwashing should be a common notion in harvesting and packing fish produce on farms [70]. Farm management may incentivize and support employees as a viable alternative to foster positive attitudes and enhance knowledge, potentially mitigating chemical-related disease outbreaks among farmers [55,71]. Employees are more inclined to adopt sustainable and healthy farming practices when they are motivated and encouraged to do so in the context of aquaculture [71]. In addition, the assistance of farm management and motivated employees may facilitate the optimization of disease control techniques and the improvement of biosecurity measures. This comprehensive methodology contributes to the enhancement of general well-being and efficiency, hence mitigating the impact of illnesses on aquaculture facilities [72]. A consensus was reached among 93% of the respondents that the act of inhaling smoking particles containing fertilizers and chemicals is harmful to one’s health. Moreover, 59% concurred that it is harmful to continue a profession without regard for safety. Therefore, shrimp farmers acknowledge the criticality of safety in their occupation and reach a consensus that proceeding without sufficient safety protocols is detrimental to one’s health. It is suggested that shrimp farming regulations incorporate occupational health and safety precautions through the adoption of the "One Health" concept, which emphasizes a holistic approach [19].

### Health safety-related practices

The practices implemented by the shrimp farmers operating in semi-intensive shrimp farms are detailed in Table 3. Despite being conscious of the need to limit health risks associated with chemicals and fertilizers through protective equipment such as masks, gloves, and other gear, fewer than half of the individuals worked with hazardous compounds. It is consistent with prior research indicating that shrimp producers were exposed to chemical-contaminated water and substances using their bare hands [32,33]. Bangladesh has implemented a Code of Conduct governing specific sectors of the shrimp farming business. This Code establishes rules and principles for various facets of shrimp farming, including environmental stewardship and resource utilization [73]. One key area of focus within the Code is the safe handling of chemicals, which directly relates to hygiene practices in shrimp farms. By outlining clear chemical usage and sanitation guidelines, the Code seeks to promote safer working conditions for shrimp farmers and ensure compliance with occupational health and safety standards. Nevertheless, consistent monitoring is necessary to assess how effectively shrimp farmers adhere to these established chemical handling practices. For instance, while the Code provides a framework, evaluating its practical implementation on the ground is crucial. Additionally, as shrimp farming in Bangladesh evolves, this Code may need to be revised in light of international norms and research [32,33,52,54,55,63,72]. Furthermore, efforts have been made to evaluate and augment adherence to global benchmarks, including the FAO Code of Conduct for Responsible Fisheries, in Bangladesh’s shrimp farming sector [30]. These efforts aim to align Bangladesh’s practices with international standards, emphasizing environmental management and occupational health safety measures such as proper sanitization and the use of personal protective equipment in aquaculture. Over 90% of shrimp farmers wash their face, hands, and legs after working on farms. Additionally, 97% of farmers demonstrated an awareness of hand hygiene before consuming food prepared with pesticides and chemicals. On the contrary, according to one study, almost 60% of the farmers consistently engaged in hand hygiene following sneezing and coughing [70]. 87% of the participants took the necessary precautions when handling raw feeds.

After using pesticides, fertilizers, and chemicals, most respondents (96%) reported rinsing their vehicles and transportation equipment with clean water. While this demonstrates a proactive approach to maintaining cleanliness, it is important to consider the effectiveness of this method in fully removing chemical residues. Research indicates that while rinsing with water can reduce surface contaminants, it may not be sufficient to eliminate all residues, particularly for water-resistant chemicals, or adhere strongly to surfaces [52,61]. More advanced cleaning techniques, such as using specific detergents or neutralizing agents, may be required to ensure thorough decontamination. Additionally, the environmental implications of this practice warrant further attention. Rinsing equipment with clean water could result in the runoff of chemical residues into surrounding water bodies, potentially contributing to pollution and harming aquatic ecosystems [63]. Therefore, while rinsing vehicles and equipment is a positive step toward maintaining hygiene, further measures—such as implementing controlled rinsing areas or using eco-friendly cleaning agents—may be necessary to minimize environmental impact and enhance the effectiveness of cleaning procedures in shrimp farming. Semi-intensive shrimp farmers would benefit from sufficient health safety training to mitigate the occurrence of illnesses associated with chemicals and pesticides and foster a more favorable perception of implementing health safety knowledge. They held the belief that by relying on reliable sources, they might mitigate any health hazards. According to prior findings (Table 2), people obtain this knowledge most frequently from their families and communities. Nonetheless, authoritative sources must furnish authentic information. They must establish trust with the farmers before imparting any knowledge. Prior to anything else, they must ascertain which information sources are reliable and readily available to these farmers. Additionally, they must ascertain the means of communication (including tools and languages) readily available to these farmers. Moreover, they must organize effective training programs to ensure that shrimp farmers not only comprehend the correct methods of shrimp production but are also aware of the critical health and safety measures that affect the overall quality of shrimp farms. Thus, the health of shrimp farmers is a significant determinant of shrimp production.

### KAP domain

We have also calculated the correlation in the KAP domain. The correlation of knowledge, attitudes, and practices is presented in Table 4. A significant positive correlation was found between knowledge and attitudes (*r_s_* = 0.425, *p*< 0.001), knowledge and practice (*r_s_* = 0.335, *p*< 0. 001) and attitude and practice (*r_s_* = 0.281, *p*< 0. 001). Furthermore, a relationship between these three health and safety domains is evident. The correlation coefficients indicated that the degree of knowledge, attitude, and practice among semi-intensive shrimp farmers were positively correlated. Similarly, studies examining the attitudes and knowledge of shrimp farmers about the use of Good Aquaculture Practices underscore the need to foster comprehension to advance constructive methodologies [71]. According to the findings, a favorable and well-informed attitude toward best practices in shrimp farming appears to facilitate their application [55,71]. Understanding and implementing the connection between knowledge, attitudes, and practices is vital to shrimp farms’ sustainable and effective operation. Acknowledging the significance of favorable attitudes and actions alongside knowledge is critical, as it fosters a sanitary environment and safeguards the welfare of both consumers and farmers. Research has shown that positive attitudes toward adopting good aquaculture practices can significantly influence the implementation of best practices in shrimp farming [71].

**Table 4:** Correlation among knowledge, attitude, and practice levels.

Level	Correlation Coefficient	P-value
Knowledge- Attitude	0.425	<0.001
Knowledge- Practice	0.335	<0.001
Attitude- Practice	0.281	<0.001

**Fig 2:**
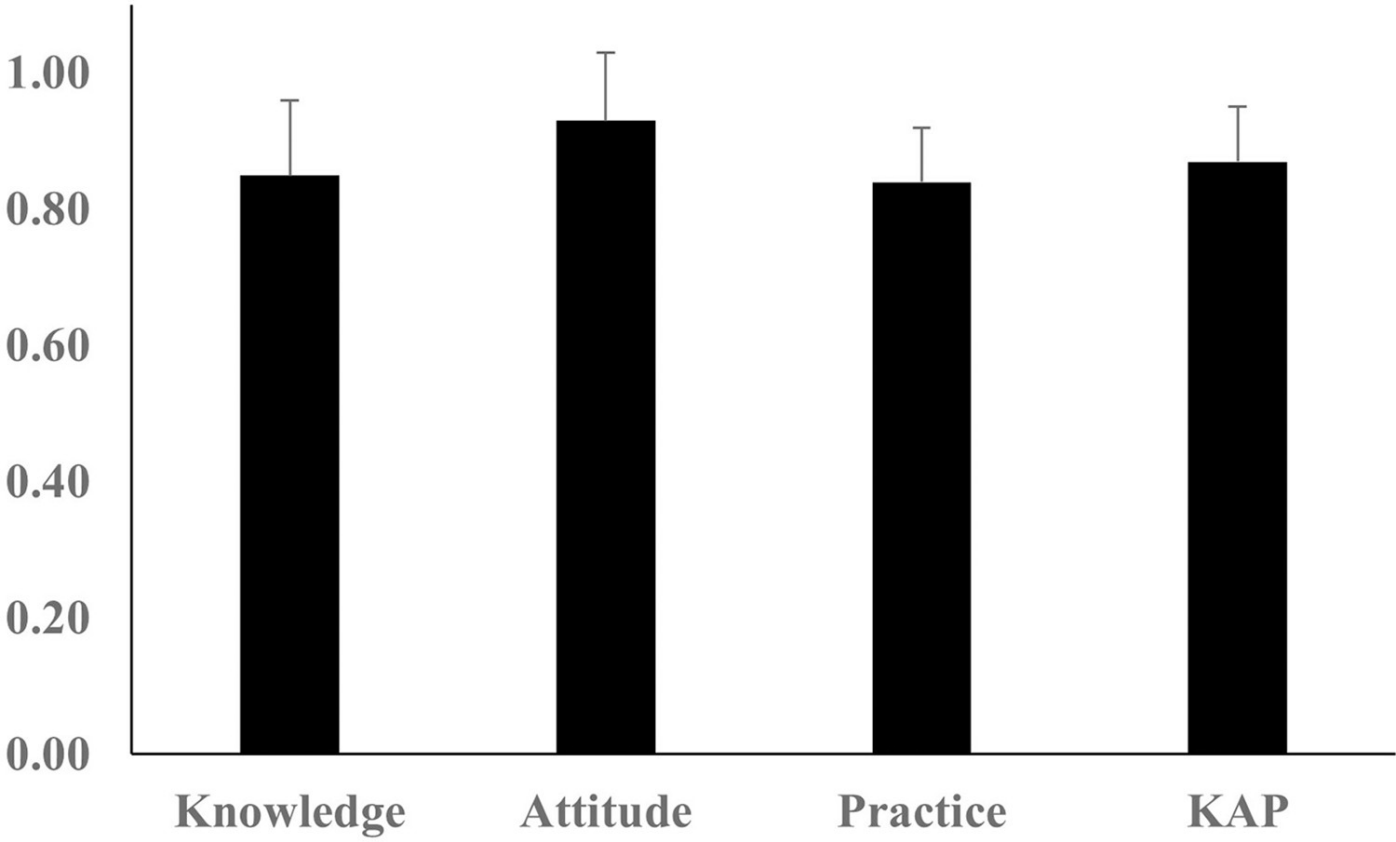
Mean and standard deviation in the KAP domain.

In this study, "positive" refers to attitudes more aligned with recommended practices and a higher willingness or openness to follow health and safety guidelines. It contrasts with lower knowledge or actual practices, where farmers may lack the information or resources to implement the necessary steps for proper shrimp farming fully. The KAP domain’s mean and standard deviation are displayed in Fig 2, where attitudes scored higher than knowledge and practice. We have also found significant differences between knowledge and attitude (*p<*0.001), knowledge and practice (*p<*0.05), and attitude and practice (*p<*0.001).

The mean attitude score was significantly higher than the knowledge and practice scores, suggesting that while farmers express favorable views about health and safety measures, their knowledge of them and their implementation in daily practices may lag. This discrepancy indicates that interventions should enhance knowledge and provide resources and support to translate positive attitudes into consistent, actionable practices. The implications of these findings are crucial for health and safety interventions in shrimp farming. While a positive attitude is an important starting point, improving knowledge and practice is essential to achieving the industry’s comprehensive safety and hygiene standards.

### Associated factors

The associated variables with the three sections of KAP have also been assessed (Table 5). The variables deemed significant by univariate linear regression are only shown in Table 5. We subsequently performed multiple linear regression analyses, as described in the methods section. Adjusted R-squared value and *p*-value show the model fit for multiple linear regression analyses. The knowledge section identified only attitude and education level as significant variables. Compared to farmers who claim to have no formal education, those with a relatively higher degree of education (>Primary) have a much greater knowledge base. Moreover, for the health and safety of shrimp farmers, education and training are additional necessities [19]. Therefore, enhanced knowledge can facilitate farmers’ comprehension of the directives issued by governing bodies (even though authorities need to study how they can properly communicate with them). Regarding practices, health and safety training demonstrates significance by adopting sound practices. Farmers lacking training experience exhibit notably inferior practice rates compared to those with training experience. Since training positively impacts safety practices, expanding and improving training programs should be prioritized. It includes developing comprehensive training modules covering all critical health and safety aspects of shrimp farming. Training programs are essential for improving health and safety practices, as shown by various studies in similar contexts [74]. Policies should mandate regular and structured training sessions for shrimp farmers. It could involve creating standards and regulations that ensure all farmers receive adequate training. Effective policies and regulations are necessary to support sustainable shrimp farming practices and improve safety. Tailor interventions to address specific gaps identified through training assessments. For example, if certain practices are still inadequate despite training, focused interventions could be designed to address these issues. Addressing specific challenges and gaps in shrimp farming practices through targeted interventions can enhance safety [75]. Implement robust monitoring and evaluation frameworks to assess the effectiveness of training programs and make data-driven adjustments. It can help understand how training changes influence safety practices over time. Monitoring and evaluating training impacts can provide insights into improving health and safety practices. By focusing on these areas, policies can be developed to enhance the impact of health safety training on shrimp farming practices, ultimately leading to improved safety and sustainability in the industry.

**Table 5:** Associated factors toward knowledge, attitude, and practices.

Features		β^#^ (95% CI)		
		Model I Knowledge	Model II Attitude	Model III Practice
Gender	Male			
	Female	-0.10 (-0.21; 0.01)		
Age Group	18–25			
	26–35	-0.03 (-0.06; 0.01)		
	36–45	-0.01 (-0.06; 0.03)		
	>45	-0.03 (-0.13; 0.06)		
Education Level	>Primary			
	Primary	-0.01 (-0.05; 0.03)		
	None	-0.06 (-0. 12; -0.01)*		
Trained in health safety	Yes			
	No			-0.03 (-0.06; -0.00)*
Health safety rating in current occupation	Safe			
	Moderately Safe	-0.06 (-0.14; 0.03)		
	Unsafe	-0.02 (-0. 20; 0.16)		
Toilet facilities	Open Space			
	Semi-pucca	-0.04 (-0.13; 0.04)		
	Sanitary/pucca	0.01 (-0.06; 0.09)		
Knowledge			0.35 (0.24; 0.47)***	0.06 (-0.05; 0.16)
Attitude		0.50 (0.33; 0.67)***		0.37 (0.25; 0.49) ***
Practice		0.12 (-0.10; 0.34)	0.50 (0.34; 0.67)***	
Adjusted R-squared		0.372	0.415	0.296
p-value ()		<0.001	<0.001	<0.001

*p<0.05; ***p<0.*001*.

*β*^*#*^ *= Beta (Coefficient)*.

CI = Confidence Interval.

The table also provides insight into the relationships among the KAP domains. The significant β value of 0.35 (95% CI: 0.24; 0.47) indicates that an increase in knowledge strongly predicts a positive change in attitude. However, the β value of 0.06 (95% CI: -0.05; 0.16) is much weaker and non-significant, indicating that knowledge alone has a minimal direct effect on practice. In the case of attitude and practice, the β value of 0.50 (95% CI: 0.34; 0.67) highlights a significant relationship, suggesting that attitude plays a crucial role in driving changes in practice. The relationship between knowledge, attitude, and practice suggests that knowledge alone may not be sufficient to influence practical behaviors. In the context of shrimp farmers, while increased knowledge can positively affect attitudes, this shift in attitude is crucial for translating knowledge into actual safety practices. This idea is supported by research, which indicates that attitudes mediate knowledge and behavior. It means that knowledge must lead to a change in attitude before it can effectively influence practice [76]. For shrimp farming, improving farmers’ attitudes toward safety practices is key to bridging the gap between what they know and how they act. Designing interventions that focus on knowledge dissemination and attitude change can lead to more effective adoption of safe practices. Studies on behavior change have consistently shown that attitudinal shifts provide a necessary condition for behaviors to align with new knowledge [71]. This approach is critical because it recognizes that behavior change in farming is not just about informing people but also about reshaping their perceptions and willingness to act on that information [7].

## Limitations

This study has several limitations. The convenience sampling may have introduced selection bias, as farmers willing to participate may not represent the broader shrimp farming population. It limits the generalizability of the findings. The cross-sectional design captures data at one point, limiting the ability to infer causality. Future studies could employ longitudinal designs to assess dynamic relationships more rigorously. The structured interview process may have led to socially acceptable responses, especially in the attitude section. Detailed data on the use of chemicals in shrimp farming, such as amounts, stages of use, and safety practices, were not fully addressed. Health risks specific to women and detailed occupational hazards (e.g., skin diseases, heavy lifting) were not comprehensively explored. Regression analyses suggested associations but did not establish causality. Longitudinal studies or advanced models like SEM are recommended for future research. We have used the KAP survey model. However, future studies can incorporate the DPSIR framework to better understand the drivers of improper health and safety measures, especially within shrimp farming. Future research can explore labor laws like the Labor Law and Labor Rules 2015 to identify any gaps in safety provisions for workers [77]. The existing data can be used to speculate how the lack of coordination among key organizations, such as the Department of Fisheries, Ministry of Labor and Employment, ILO, and UNIDO, could contribute to the observed challenges. Future studies should investigate this in more detail to propose viable solutions. Nevertheless, this study provides a nascent avenue for further inquiry into this neglected facet of the fisheries sector since most academics in Bangladesh have concentrated only on shrimp production (not the farmers). Additionally, it has the potential to aid policymakers in the public health and fisheries sectors in their quest for knowledge regarding shrimp farming. Additionally, they may organize a variety of training exercises better to equip the shrimp farmers with the necessary skills.

## Conclusion

Respondents (semi-intensive shrimp farmers) exhibited favorable levels of knowledge and attitudes toward health and safety during the use of chemicals, pesticides, and fertilizers in semi-intensive shrimp farms, according to the study’s findings. However, several tasks remain unpracticed despite their knowledge and favorable attitudes. They could lack the necessary resources and infrastructure to implement those practices. Alternatively, individuals may resist adhering to such directions as wearing gloves and masks while handling fertilizers and chemicals. Furthermore, many shrimp farmers lacked official health and safety training. Workers’ responses in semi-intensive shrimp farming indicate excellent health and high job satisfaction. Nevertheless, the semi-intensive shrimp farmers did not understand health and safety protocols. Ensuring workers have safe and healthy working conditions is another critical factor in maintaining a consistent shrimp supply. Including occupational health considerations in creating technologies may lower the risk of sickness for technology providers. It may be accomplished with the participation of semi-intensive shrimp farmers and methodical design procedures. Enforcement of rules and inspections of semi-intensive shrimp farms are critical responsibilities of the authorities. Therefore, the government and other relevant regulatory agencies should implement systematic and effective management methods and stringent restrictions for certification, training, law, and legislation to guarantee the safety of programs. Additionally, improved extension services and training would aid in raising farmers’ health consciousness and reducing the incidence of illnesses caused by fertilizers and chemicals. Extension and farmer education are comparatively economical approaches to enhancing production efficiency.

## Supporting information

S1 Dataset Dataset has been attached. (XLSX)
